# Inflammation-regulating factors in ascites as predictive biomarkers of drug resistance and progression-free survival in serous epithelial ovarian cancers

**DOI:** 10.1186/s12885-015-1511-7

**Published:** 2015-07-01

**Authors:** Denis Lane, Isabelle Matte, Perrine Garde-Granger, Claude Laplante, Alex Carignan, Claudine Rancourt, Alain Piché

**Affiliations:** 1 Département de Microbiologie et Infectiologie, Faculté de Médecine, Université de Sherbrooke, 3001, 12ième Avenue Nord, J1H 5 N4 Sherbrooke, Canada; 2 Département de Pathologie, Faculté de Médecine, Université de Sherbrooke, 3001, 12ième Avenue Nord, J1H 5 N4 Sherbrooke, Canada

**Keywords:** Ascites, Ovarian cancer, Tumor microenvironment, Cytokines, Inflammation, Drug resistance

## Abstract

**Background:**

Platinum-based combination therapy is the standard first-line treatment for women with advanced serous epithelial ovarian carcinoma (EOC). However, about 20 % will not respond and are considered clinically resistant. The availability of biomarkers to predict responses to the initial therapy would provide a practical approach to identify women who would benefit from a more appropriate first-line treatment. Ascites is an attractive inflammatory fluid for biomarker discovery as it is easy and minimally invasive to obtain. The aim of this study was to evaluate whether six selected inflammation-regulating factors in ascites could serve as diagnostic or drug resistance biomarkers in patients with advanced serous EOC.

**Methods:**

A total of 53 women with stage III/IV serous EOC and 10 women with benign conditions were enrolled in this study. Eleven of the 53 women with serous EOC were considered clinically resistant to treatment with progression-free survival < 6 months. Ascites were collected at the time of the debulking surgery and the levels of cytokines were measured by ELISA. The six selected cytokines were evaluated for their ability to discriminate serous EOC from benign controls, and to discriminate platinum resistant from platinum sensitive patients.

**Results:**

Median ascites levels of IL-6, IL-10 and osteoprotegerin (OPG) were significantly higher in women with advanced serous EOC than in controls (*P* ≤ 0.012). There were no significant difference in the median ascites levels of leptin, soluble urokinase plasminogen activator receptor (suPAR) and CCL18 among serous EOC women and controls. In Receiver Operator curve (ROC) analysis, IL-6, IL-10 and OPG had a high area under the curve value of 0.905, 0.832 and 0.825 respectively for distinguishing EOC from benign controls. ROC analysis of individual cytokines revealed low discriminating potential to stratify patients according to their sensitivity to first-line treatment. The combination of biomarkers with the highest discriminating potential was with CA125 and leptin (AUC = 0.936, 95 % CI: 0.894–0.978).

**Conclusion:**

IL-6 was found to be strongly associated with advanced serous EOC and could be used in combination with serum CA125 to discriminate benign and EOC. Furthermore, the combination of serum CA125 and ascites leptin was a strong predictor of clinical resistance to first-line therapy.

**Electronic supplementary material:**

The online version of this article (doi:10.1186/s12885-015-1511-7) contains supplementary material, which is available to authorized users.

## Background

Epithelial ovarian cancer (EOC) is the leading cause of gynecological cancer-related death [1, 2]. Serous carcinomas are the most frequent subtype encountered in patients with EOC [3]. Being largely asymptomatic, over 70 % of patients are diagnosed at an advanced stage of the disease (stage III/IV) with metastasis throughout the peritoneal cavity and large amount of ascites [1, 3, 4]. Platinum-based combination chemotherapy is the standard first-line treatement for advanced stage EOC. Although overall initial response rates to first-line platinum based chemotherapy are good, 15–20 % of patients will not respond to the initial chemotherapy [5]. The tumors are considered resistant if the patient do not respond to platinum-based therapy or show progression during the course of therapy, or if the clinical progression-free survival (PFS) is less than 6 months [6]. These patients are considered to have intrinsic resistance to first-line treatment. There is currently no available biomarker to identify these patients at baseline. Unfortunately, these patients are identified retrospectively after they experienced early relapse or did not respond to initial treatment. Thus, customised treatments and clinical stratification of these EOC patient remain critical objectives in the field. The identification of new biomarkers for intrinsic drug resistance would represent a substantial step forward in our efforts to adequately treat EOC and increase survival.

The only clinically validated biomarker for disease monitoring and assessing response and relapse to treatment is CA125 which is encoded by MUC16 mucin gene [7–12]. The N-terminal extracellular region of MUC16 is cleaved and released into the serum of patients with EOC [9]. Serum CA125 lacks specificity and sensitivity, as a single marker, for early EOC detection and prognosis [13]. Recent studies suggest that a Risk of Ovarian Malignancy Algorithm (ROMA) incorporating CA125 and HE4 levels in serum shows a high potential for discriminating ovarian cancer from benign gynecological diseases [14–16]. HE4 is the only biomarker, other than CA125, that has been approved as a diagnostic marker for ovarian cancer [17].

Tumor-promoting inflammation is now established as a hallmark of cancer [18, 19]. Serum cytokine levels have been investigated as diagnostic and prognostic markers in ovarian cancer. Ascites from women with advanced serous EOC is an inflammatory milieu rich in inflammation promoting factors. An inflammatory environment such as ascites promotes drug resistance of EOC cells [20–23]. High levels of pro-inflammatory cytokines, chemokines and growth factors are found in OC ascites [23–29]. A recent multiplex profiling of cytokines in the ascites of 10 EOC patients has demonstrated enhanced expression of several inflammation-regulating factors including IL-6, IL-6R, IL-8, IL-10, leptin, osteoprotegerin (OPG) and urokinase plasminogen activator (uPAR) among others [30]. Specific inflammatory cytokines in ascites such as IL-6 were shown to be an independent prognostic factor of worse outcome [31]. IL-6 contributes to EOC progression by inhibition of apoptosis, stimulation of angiogenesis, increased migration and invasion, and stimulation of cell proliferation [32–35].

Ascites is an attractive biofluid for biomarker discovery as it is easy and minimally invasive to obtain. Proximal fluids such as ascites – as opposed to serum – might reflect events in ovarian tumorigenesis earlier than in peripheral blood circulation [36]. Furthermore, the concentration of cytokines is usually much higher in ascites compared to serum [29]. Thus, the accessibility of ascites – a simple non-invasive puncture - provides an excellent source of inflammation promoting factors (with potential enrichment relative to serum) for the investigation of prognostic biomarkers.

Ascites from a small subset of serous EOC patients and patients with benign gynecological conditions has been previously analyzed with a panel of 120 cytokines by cytokine array [30]. This analysis has revealed 20 cytokines/growth factors, which showed a statistically significant (*P* < 0.01) > 2-fold up-regulation relative to benign fluids. For this study, six inflammatory-regulating factors including IL-6, IL-10, leptin, osteoprotegerin (OPG), soluble urokinase plasminogen activator receptor (suPAR) and CCL18 were initially selected based on the following biological rationales: 1) IL-6, IL-10, leptin, OPG, suPAR and CCL18 are present at high levels in EOC ascites [29, 30]; 2) high ascites levels of IL-6, IL-10, leptin and OPG have been associated with EOC worse outcome [30]; 3) their concentrations in ascites are well within the range required to induce a biological effect [29, 30]; 4) IL-6, IL10, leptin, suPAR and OPG can inhibit drug-induced apoptosis *in vitro* in EOC cells or other cancer cells [34, 37–46].

In the present study, we have measured the baseline levels of six inflammation-regulating factors including IL-6, IL-10, leptin, OPG, suPAR and CCL18 in prospectively collected ascites patients with advanced serous EOC with complete clinicopathologic data and adequate follow up. The aims of the study was to establish (1) whether levels of these cytokines differ between benign and serous EOC, (2) whether levels can distinct patients with intrinsic drug resistance to those that respond to first-line platinum-based treatment.

## Methods

### Patients

Ascites is routinely obtained at the time of the debulking surgery of ovarian cancer patients treated at the Centre Hospitalier Universitaire de Sherbrooke. After collection, cell-free ascites are stored at - 80 °C in our tumor bank until use. The study population consisted of 53 women with newly diagnosed epithelial ovarian cancer admitted at the Centre Hospitalier Universitaire de Sherbrooke. Ten cases with benign conditions, namely histologically benign gynecological conditions including fibromas (5), mucinous and serous cystadenomas (4), and one inflammatory lesion, constituted the control group. This study was approved by the Institutional Review Board of the Centre de Recherche Étienne-Le Bel. Informed consent was obtained from women that underwent surgery by the gynecologic oncology service between 2000 and 2013. All samples were reviewed by an experienced pathologist. Baseline characteristics and serum CA125 levels were collected for all patients. All patients had a follow up ≥ 12 months. Disease progression was defined by either serum CA125 ≥ 2 X nadir value on two occasions, documentation of lesion progression or appearance of new lesions on CT-scan or death [37]. Patient’s conditions were staged according to the criteria of the International Federation of Gynecology and Obstetrics (FIGO). PFS was defined by the time from the initial surgery to evidence of disease progression. Drug resistance was defined as those with PFS < 6 months or lack of response to initial platinum-based chemotherapy. Patient characteristics are summarised in Table 2.

### Peritoneal fluid specimens

Peritoneal fluids and ascites were obtained at the time of initial cytoreductive surgery for all patients. Peritoneal fluids were centrifuged at 1000 rpm for 15 min and cell-free supernatants were stored at−80 °C until assayed. All acellular fluids were supplied by the Banque de tissus et de données of the Réseau de Recherche en Cancer of the Fonds de la Recherche du Québec en Santé affiliated to the Canadian Tumor Repository Network (CTRNet).

### ELISA measurements

Cytokine levels in peritoneal fluid samples were determined by ELISA using the commercially available human Quantikine kits from R&D Systems (Minneapolis, MN). OPG levels were determined using an ELISA from E Bioscience (Vienna, Austria). The assays were performed in duplicate according to the manufacturer’s protocols. The detection thresholds were 0.79 pg/ml for IL-6, 2.9 pg/ml for IL-10, 7.8 pg/ml for leptin, 4.5 pg/ml for OPG, 33 pg/ml for suPAR and 1.1 ng/ml for CCL18. The intra-assay variability was 5–10 % for IL-6, 2.5–6.6 % for IL-10, 3–3.2 % for leptin, 4.3–7.9 % for OPG, 2.1–7.5 % for suPAR and 3.2–3.7 % for CCL18. The inter-assay variability varied from 3.5 to 7.6 % depending on the cytokine. All samples were examined in duplicate and the median values were used for statistical analysis.

### CA125 measurements

CA125 was determined at Centre Hospitalier Universitaire de Sherbrooke laboratory in serum samples by EIA using the Elecsys 2010 analyzer and CA125 II regents (Roche Diagnostics, Québec, Canada). The reference range was 0–35 kUI/L.

### Statistical analysis

Comparison between unpaired groups was made using the Mann–Whitney test or the Kruskal-Wallis test. Statistical differences in PFS were determined by the log-rank test, and Kaplan-Meier survival curves were made. PFS was defined as the interval between the date of the initial debulking surgery and the time of disease progression or the last date of follow up. Receiver-operator curves (ROC) were created to determine the predictive value of the cytokines to distinguish between EOC patients and control, and between clinically resistant and sensitive patients. The threshold for statistical significance is *P* < 0.05.

## Results

### Predictive value of ascites inflammation-regulating factors for EOC versus control group

**Fig. 1:**
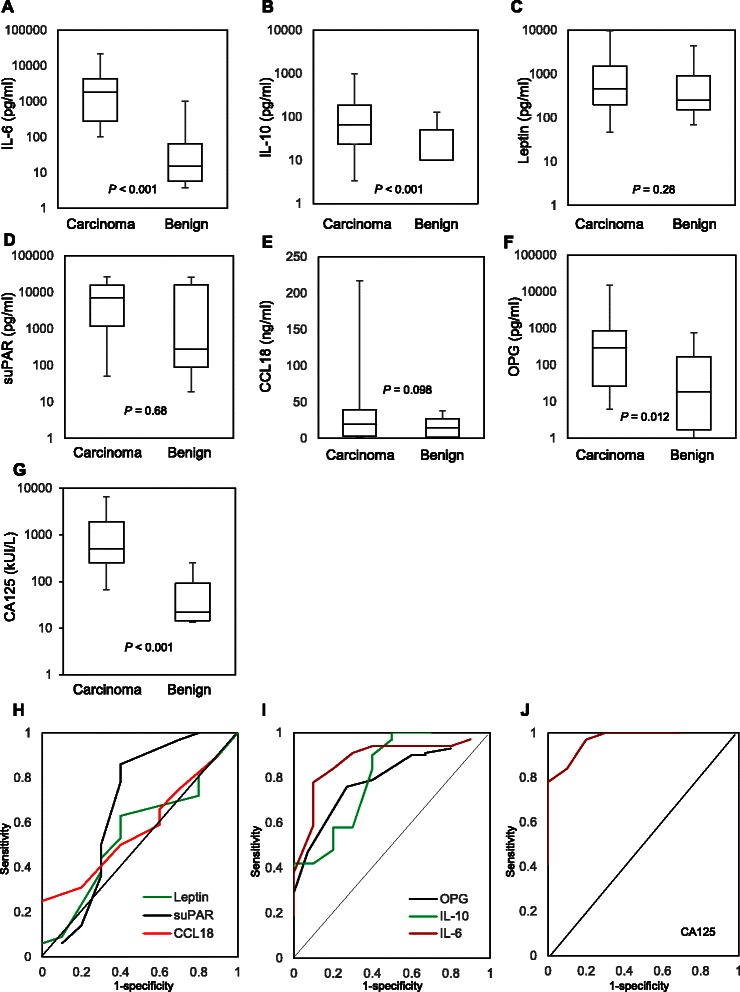
Ascites levels of inflammation-regulating factors in serous EOC patients and those with benign conditions. Box plots representing ascites levels of IL-6 (**a**), IL-10 (**b**), leptin (**c**), suPAR (**d**), CCL18 (**e**) and OPG (**f**) in patients with advanced serous EOC and patients with benign gynecological conditions. (**g**) Box plot of serum CA125 levels in serous EOC patients and patients with benign gynecological diseases. The *P* value is indicated for each factor. ROC analysis using leptin, suPAR and CCL18 (**h**), and IL-6, IL-10 and OPG (**i**) for distinguishing patients with serous EOC from control patients. (**j**) ROC analysis of serum CA125 for distinguishing serous EOC from control patients

Expression levels of IL-6, IL-10, leptin, OPG, suPAR and CCL18 in ascites were measured by ELISA. These inflammation-regulating factors were measured in a cohort of 53 patients with advanced (stage III/IV) serous EOC from ascites that were obtained at the time of their debulking surgery. Median IL-6 ascites levels were 121-fold, IL-10 levels 9.8-fold and OPG levels 16.4-fold higher in serous EOC samples compared to benign controls (Table 1, Fig. 1a, b and f). In contrast, median CCL18 and leptin ascites levels were not statistically different in serous EOC compared to benign controls (Table 1, Fig. 1c and e). Although, median levels of suPAR were almost 29-fold higher in serous EOC patients, the difference was not statistically significant (*P* = 0.68) (Table 1, Fig. 1d). IL-6 and IL-10 levels were undetectable in 6 % of serous EOC and in 10 % and 40 % of the benign controls respectively. Serum CA125 levels were measured and the median level was 23-fold higher in serous EOC sample compared to control with a *P* < 0.001 (Fig. 1g). The expression of IL-6 in the ascites of serous EOC patients did not show a strong correlation with those of IL-10 (correlation coefficient, *R* < 0.1). We also observed a lack of significant correlation between the expression of IL-6 and those of leptin, suPAR and CCL18 with *R* < 0.1.

**Table 1:** Ascites levels of the selected inflammatory cytokines

Cytokines	Benign controls median, pg/ml	Serous EOC median, pg/ml	Fold change (FC) relative to benign	*P* value
IL-6	15 (6–65)	1820 (279–4327)	121	<0.001
IL-10	10 (0–45)	97,5 (23–186)	9.8	<0.001
Leptin	254 (152–917)	453 (177–1956)	1.8	NS
suPAR	272 (89–15,944)	7021 (1170–15,538)	28.8	NS
CCL18	15,000 (2000–27,000)	20,000 (3000–39,000)	1.3	NS
OPG	18 (2–166)	296 (23–865)	16,4	0.012

Values in brackets indicate 25–75 quartiles

*NS* not statistically significant

*P* value = Student *T* test

**Fig. 2:**
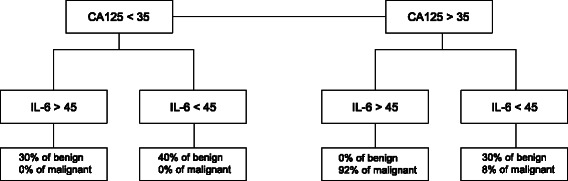
Serum CA125 and ascites IL-6 levels can discriminate between patients with serous EOC or benign gynecological conditions. The markers with cutoff (pg/ml for IL-6 and kUI/L for CA125) are depicted together with the percentage of the patients with EOC or benign conditions that were predicted by the combination of markers

ROC analyses were performed to determine the predictive value of ascites factors distinguishing EOC patients from the control group. Ascites levels of IL-6 allowed most accurate discrimination (AUC = 0.905, 95 % CI: 0.850–0.960) between EOC patients and benign controls although it did not outperformed serum CA125 (AUC = 0.951, 95 % CI: 0.906–0.996) (Fig. 1i and j). IL-10 and OPG also discriminated serous EOC patients from benign controls with AUC = 0.832 (95 % CI: 0.763–0.901) and AUC = 0.825 (95 % CI: 0.782–0.868 respectively (Fig. 1j). The other inflammation-regulating factors tested had lower discriminating potential with AUC for suPAR = 0.757 (95 % CI: 0.632–0.882), for leptin = 0.586 (95 % CI: 0.488–0.684) and for CCL18 = 0.612 (95 % CI: 0.538–0.686) (Fig. 1h). The results did not reach statistical significance for suPAR, leptin and CCL18. Thus, ascites levels of IL-6 in this study proved to be the most reliable cytokine biomarker for discriminating EOC serous patients from the control group. At a cutoff value of 75 pg/ml for IL-6, the sensitivity was 92 % and the specificity was 80 %. Combining CA125 and IL-6 further improved specificity. In patients with serum levels above the cutoff point of CA125 > 35 kUI/L, a cutoff point of IL-6 > 45 pg/ml gave a specificity of 100 % for distinguishing between EOC and control group (Fig. 2).

### Discriminating potential of ascites inflammation-regulating factors to identify women with intrinsic drug resistance

Inflammation has been associated with tumor progression and drug resistance [18, 19]. Serous EOC ascites has been previously shown to inhibit drug-induced apoptosis [20–23]. Inflammation-regulating factors may enhance cisplatin resistance [32–35, 42, 44, 46]. ROC were created to determine the predictive value of ascites IL-6, IL-10, leptin, OPG, suPAR and CCL18 for discriminating, at baseline, clinically resistant patients from those that are sensitive. The clinical and pathological characteristics of the patients in our cohort are shown in Table 2. Of the 53 patients, 42 were drug sensitive and 11 were drug resistant. The median age at diagnosis was 60 years (range, 27 to 85 years), and all patients had advanced-stage (FIGO stages III/IV) with serous histology. Most (≥79 %) of patients were optimally cytoreduced after initial surgery, and about 30 % received pre-operative chemotherapy. There was no significant difference between the two groups. All patients had a follow-up ≥ 12 months (range, 12 to 108 months). Clinically sensitive patients have a median PFS of 13.9 months and clinically resistant patients a median PFS of 4 months.

**Table 2:** Patient characteristics

Characteristic	Drug sensitive patients	Drug resistant patients	*P* value
*n* = 53	(*n* = 42)	(*n* = 11)	
Age (years)			NS
Median	61,5	62	
Range	31–81	27–89	
FIGO stage			NS
I–II	0 (0)	0 (0)	
III–IV	42 (100)	11 (100)	
Grade			NS
1	4 (10.5)	0 (0)	
2	8 (21)	2 (18)	
3	19 (50)	8 (73)	
ND	0 (0)	1 (9)	
Histologic subtype			NS
Serous	42 (100)	11 (100)	
Debulking status			NS
<2 cm	33 (79)	9 (82)	
>2 cm	5 (12)	2 (18)	
ND	4 (10)	0 (0)	
Prior chemotherapy			NS
Yes	9 (21)	4 (36)	
No	33 (79)	7 (64)	
CA125 at diagnosis			NS
Median	626	1145	
Range	20–6549	88–14,180	

*FIGO* international federation of gynecology and obstetrics, *NS* not statistically significant, *ND* not determined

**Fig. 3:**
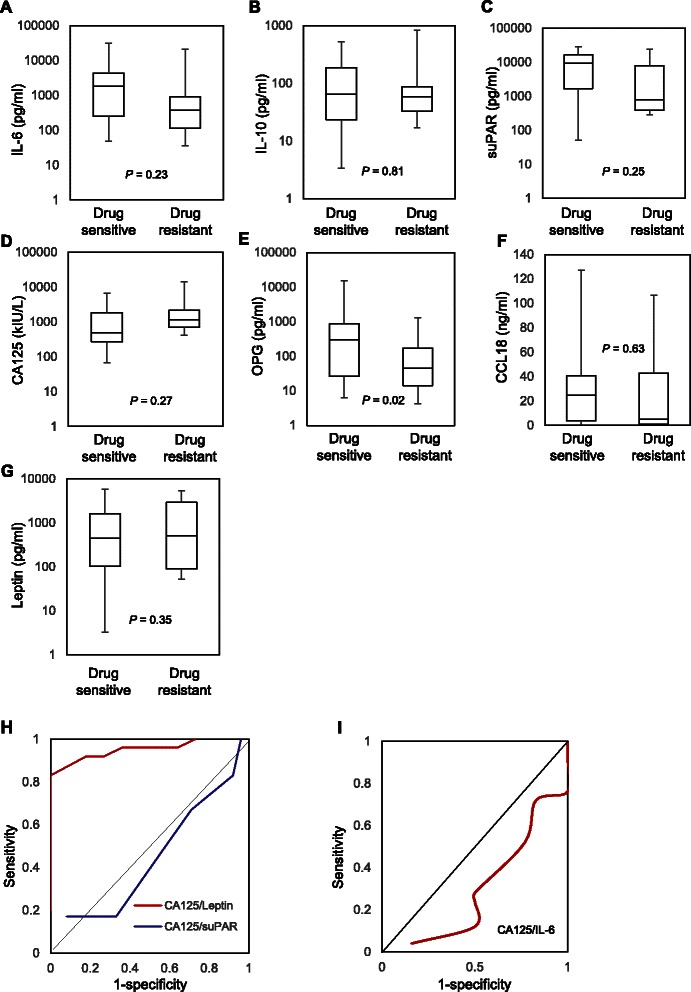
Ascites levels of inflammation-regulating factors in clinically resistant patients and those sensitive to first-line treatment. Box plots representing ascites levels of IL-6 (**a**), IL-10 (**b**), suPAR (**c**), serum CA125 (**d**), OPG (**e**), CCL18 (**f**) and leptin (**g**) in patients with resistance to first-line therapy and patients with sensitive diseases. The *P* value is indicated for each factor. ROC analysis using the combination of CA125/leptin and CA125/suPAR (**h**) and CA125/IL-6 (**i**) for distinguishing patients with resistant or sensitive EOC

Median ascites levels of IL-6 and IL-10, and serum levels of CA125, were not statistically different between patients that had drug sensitive or drug resistant diseases (Fig. 3a-c). Similarly, median levels of leptin, suPAR and CCL18 were not significantly different (data not shown). In contrast, ascites OPG levels were significantly higher in chemosensitive patients compared to resistant patients (Fig. 3d). ROC analysis for individual cytokines revealed low discriminating potential to stratify patients according to their sensitivity to first-line treatment (Additional file 1: Figure S1). To improve the accuracy, we assessed combinations of the studied cytokines and CA125 in ROC analysis. The combination of biomarkers with the highest discriminating potential was with CA125 and leptin (AUC = 0.936, 95 % CI: 0.894–0.978) (Fig. 2d). All other combination, including CA125 with suPAR (Fig. 3d) and CA125 with IL-6 (Fig. 3e), had low discriminating potential with AUC < 0.650.

### Inflammation-regulating factor levels as prognostic marker in serous EOC

**Fig. 4:**
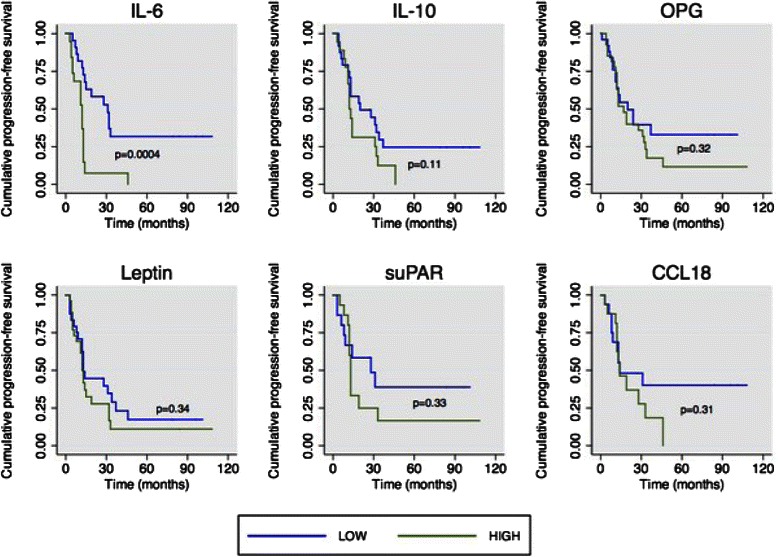
Kaplan-Meier curves of ascites IL-6, IL-10, OPG, leptin, suPAR and CCL18. The median levels of each factor were taken as cutoff points. The *P* value is indicated for each factor

We assessed the prognostic value of IL-6, IL-10, leptin, OPG, suPAR and CCL18 in relation with PFS in the cohort of 53 patients. A cutoff value corresponding to the median of each factor was used to separate patients into two groups: those with high ascites levels versus those with low ascites levels. Kaplan-Meier curves of the six factors are shown in Fig. 4. Among the six inflammation-regulating factors, only IL-6 was significantly associated with a worse outcome. Patients with low ascites IL-6 levels had a median PFS of 12 months compared to patients with high levels who had a PFS of 28 months (*P* = 0.0004, log rank test).

## Discussion

We selected for this study patients with advanced serous EOC to ensure a homogenous group of patients and because this subtype is the most frequently encountered subtype in clinic. In this context, the conclusions of this study may not apply to other ovarian cancer sub-types or to patients presenting with FIGO stage I/II diseases. However, this study has the advantage of comprising a homogeneous group of women with advanced serous EOC, thus limiting potential bias associated with inclusion of various sub-types with distinct genetic backgrounds. In our study, ascites levels of IL-6, IL-10 and OPG were found to be elevated in patients with advanced stage serous EOC compared with patients with benign gynecological conditions. Moreover, determination of IL-6 levels could classify 68 % of the advanced stage serous EOC patients accurately, without falsely classifying patients with benign gynecological conditions. These findings are in line with previous studies demonstrating higher levels of IL-6, IL-10 and OPG in malignant ascites or serum compared to patients with benign conditions [29, 47, 48]. In a recent study, IL-6 levels in ascites were the most discriminating to distinguish EOC patients from patients with benign conditions among ten selected factors [49]. Without surprise, serum CA125 levels were found to be the most discriminating factor for advanced stage serous EOC patients. Indeed, CA125 was elevated (>35 kUI/L) in 100 % of EOC patients and in 30 % of patients with benign conditions in this study. Others found CA125 commonly elevated in serous EOC patients but it has not always consistently discriminated between malignant and benign pelvic mass [50]. Serum CA125 may be elevated in a variety of other benign conditions [17, 50]. Therefore, CA125 alone lacks specificity. Our data suggest that ascites IL-6 might be a good addition to serum CA125 for diagnosis of serous EOC versus benign conditions. In our study, a cutoff point of CA125 > 35 kUI/L and a cutoff point of IL-6 > 45 pg/ml gave a sensitivity of 92 % and a specificity of 100 % for distinguishing between EOC and control group. One limitation of this study is that data were derived from a small number of samples, thus conclusions should be viewed appropriately. Further studies however are needed to evaluate the additional value of ascites IL-6 in combination with serum CA125 to discriminate advanced stage serous EOC patients and patients with benign gynecological conditions. Indeed, because of its retrospective nature, a confirmation of our results in a larger cohort is necessary.

IL-6 production generates an inflammatory environment that promotes metastatic growth. In this context, there is a number of studies that linked serum or ascites IL-6 levels with a worse prognosis and poor overall survival in EOC patients [31, 51, 52]. In line with these studies, our data demonstrate that higher IL-6 levels were significantly associated with shorter PFS. In addition, IL-6 has been associated, in some context, with cisplatin resistance *in vitro* through upregulation of anti-apoptotic proteins, such as Bcl-2 and IAPs, and downregulation of pro-apoptotic proteins, such as BID and BAX [34, 53]. In this study however, we did not observed a correlation between IL-6 levels in ascites and clinical resistance to cisplatin. Furthermore, using IL-6 concentrations (500 to 5000 pg/ml) at levels similar to those found in ascites, we have found no effect on cisplatin-induced cell death in EOC cell lines (data not shown). IL-6 does however promotes cell migration and invasion *in vitro* as such may contribute to metastatic growth and worse prognosis.

The second goal of the study was to determine if a single inflammation-regulating factor, or a combination of factors, could be used as a predictive value to discriminate clinically resistant versus sensitive patients. This is critical because the prognosis of women with EOC is strongly associated with the length of PFS after first-line therapy [54]. The availability of biomarkers to predict responses to the initial therapy would provide a practical approach to identify women who would benefit from a more appropriate first-line treatment. Because ascites is a proinflammatory milieu rich in cytokines, chemokines and growth factors, and because ascites may enhance resistance to various drugs, it constitutes an excellent reservoir for the identification of drug resistance biomarkers. There is a large effort in the field of EOC to identify new diagnostic and prognostic biomarkers, in particular for clinically resistant patients [55–57]. Huang et al. have performed proteomic studies of ovarian cancer ascites using gel electrophoresis coupled with matrix-assisted laser desorption/ionization time-of-flight mass spectrometry, and compared chemoresistant and chemosensitive patients [55]. They found that ceruloplasmin levels, an acute phase protein, was significantly higher in chemoresistant than in chemosensitive ascites. Such acute phase protein levels are often modulated by chemotherapy treatments [58]. Therefore, ceruloplasmin may act not as a causal protein but as a marker of systemic inflammation. In ROC analysis, the combination of CA125 and leptin had the highest discriminating potential (AUC 0.936) to distinguish clinically resistant patients to first-line therapy from sensitive patients presenting with advanced serous EOC.

Interestingly, CA125 expression has been associated with resistance to cisplatin and death receptor ligand in ovarian and breast cancer cell lines [59–61]. It was suggested that CA125 affects tumor cells by altering the expression of pro- and anti-apoptotic proteins [59, 61]. Leptin has been shown to activate PI3K/Akt and ERK1/2 survival pathways and stimulate the expression of anti-apoptotic protein Mcl-1 in ovarian cancer cell line OVCAR3 [62]. Furthermore, serous EOC ascites was found to activate PI3K/Akt and ERK1/2 pathways and stimulate the expression of Mcl-1 in ovarian cancer cells [20, 22]. These signaling alterations were associated with increased resistance to death receptor-induced apoptosis. Altogether, these data provide a biological rationale for the findings that the combination of CA125 and leptin discriminate between sensitive and resistant patients.

## Conclusions

In conclusion, ascites IL-6 was found to be strongly related to serous EOC and may be used in combination with CA125 for diagnosis of advanced serous EOC. This finding however requires further validation. Serum CA125 in combination with leptin has the potential to discriminate clinically resistant from sensitive patients at baseline and could therefore be used to stratify patients at baseline that are more likely to benefit from standard first-line treatment among patients presenting with advanced serous EOC. The potential role of CA125 and leptin needs to be further explored.

## Additional file

Additional file 1: Figure S1. Receiver operator curve (ROC) analysis by using single inflammation-regulating factor to differentiate patients resistant to first-line treatment (PFS < 6 months) from those that are clinically sensitive to first-line treatment (PFS > 6 months).
