# Supplementary material for: Inflammation-regulating factors in ascites as predictive biomarkers of drug resistance and progression-free survival in serous epithelial ovarian cancers
Source: BMC Cancer. 2015 Jul 1;15:492. doi: 10.1186/s12885-015-1511-7 (PMC4486134; doi:10.1186/s12885-015-1511-7)
Supplement: Additional file 1: Figure S1. — Receiver operator curve (ROC) analysis by using single inflammation-regulating factor to differentiate patients resistant to first-line treatment (PFS < 6 months) from those that are clinically sensitive to first-line treatment (PFS > 6 months). [file 12885_2015_1511_MOESM1_ESM.ppt]

## Slide 1
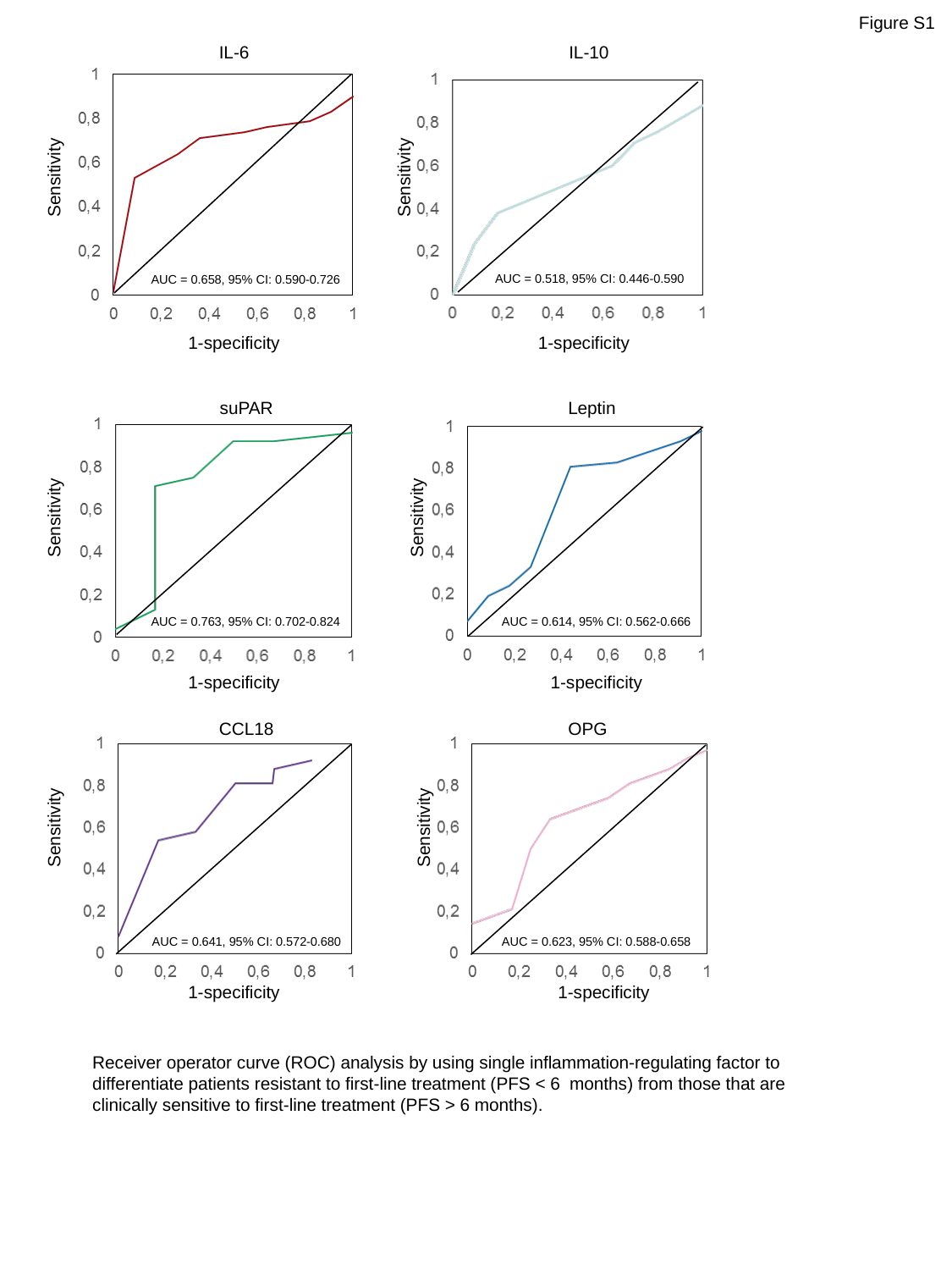

Figure S1
IL-6
IL-10
Sensitivity
Sensitivity
AUC = 0.518, 95% CI: 0.446-0.590
AUC = 0.658, 95% CI: 0.590-0.726
1-specificity
1-specificity
suPAR
Leptin
Sensitivity
Sensitivity
AUC = 0.763, 95% CI: 0.702-0.824
AUC = 0.614, 95% CI: 0.562-0.666
1-specificity
1-specificity
CCL18
OPG
Sensitivity
Sensitivity
AUC = 0.641, 95% CI: 0.572-0.680
AUC = 0.623, 95% CI: 0.588-0.658
1-specificity
1-specificity
Receiver operator curve (ROC) analysis by using single inflammation-regulating factor to
differentiate patients resistant to first-line treatment (PFS < 6 months) from those that are
clinically sensitive to first-line treatment (PFS > 6 months).

## Slide 2
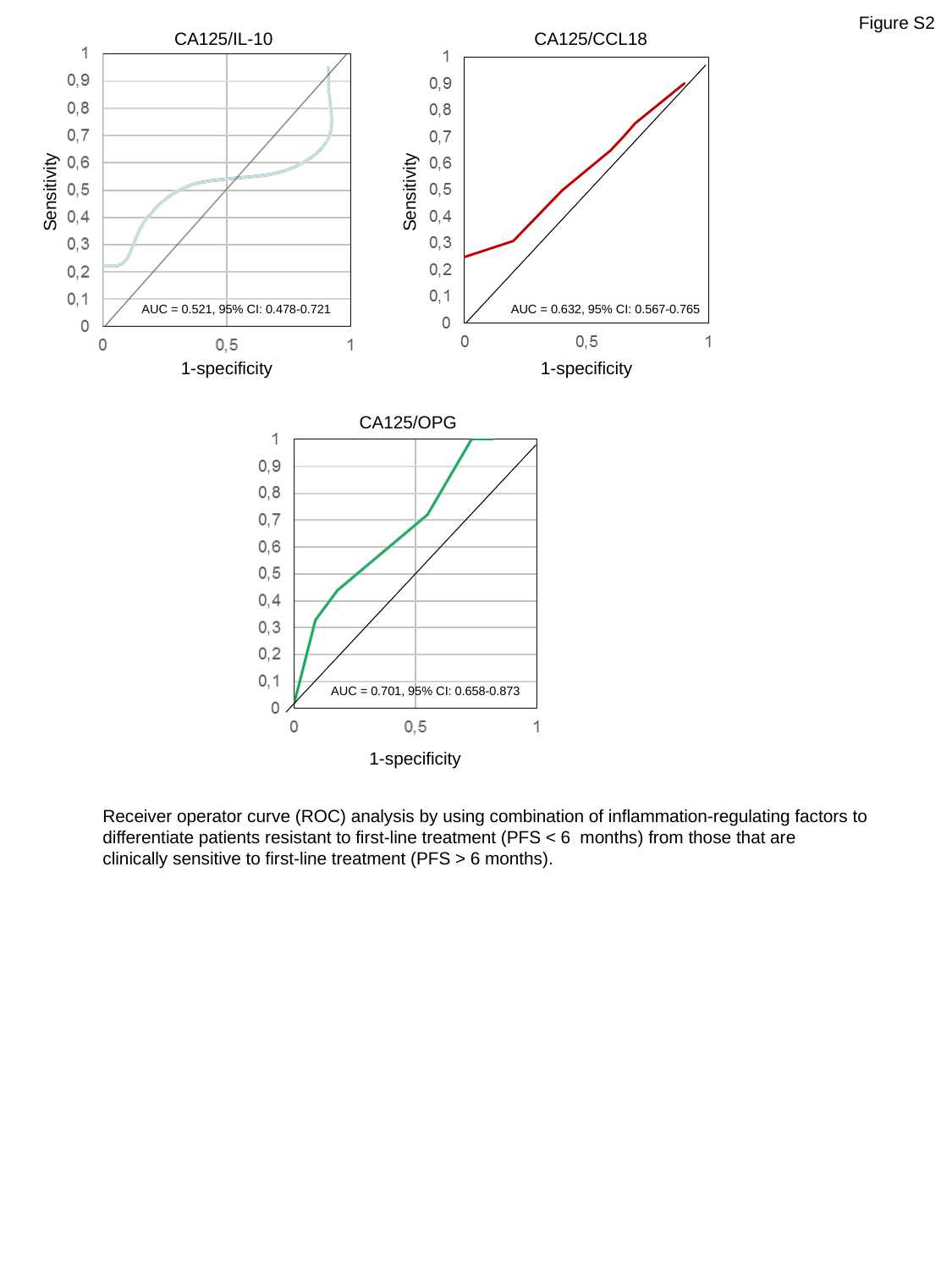

Figure S2
CA125/IL-10
CA125/CCL18
Sensitivity
Sensitivity
AUC = 0.521, 95% CI: 0.478-0.721
AUC = 0.632, 95% CI: 0.567-0.765
1-specificity
1-specificity
CA125/OPG
AUC = 0.701, 95% CI: 0.658-0.873
1-specificity
Receiver operator curve (ROC) analysis by using combination of inflammation-regulating factors to
differentiate patients resistant to first-line treatment (PFS < 6 months) from those that are
clinically sensitive to first-line treatment (PFS > 6 months).
